# Challenges and perspective of drug repurposing strategies in early phase clinical trials

**DOI:** 10.18632/oncoscience.173

**Published:** 2015-06-30

**Authors:** Shumei Kato, Stacy L. Moulder, Naoto T. Ueno, Jennifer J. Wheler, Funda Meric-Bernstam, Razelle Kurzrock, Filip Janku

**Affiliations:** ^1^ Department of Investigational Cancer Therapeutics, The University of Texas MD Anderson Cancer Center, Houston, Texas, USA; ^2^ Department of Breast Medical Oncology, The University of Texas MD Anderson Cancer Center, Houston, Texas, USA; ^3^ Center for Personalized Cancer Therapy and Division of Hematology and Oncology, Department of Medicine, UC San Diego Moores Cancer Center, La Jolla, California, USA

**Keywords:** Drug repurposing, early phase trial, cancer, drug development

## Abstract

Despite significant investments in the development of new agents only 5% of cancer drugs entering Phase I clinical trials are ultimately approved for routine clinical cancer care. Drug repurposing strategies using novel combinations of previously tested anticancer agents could reduce the cost and improve treatment outcomes. At MD Anderson Cancer Center, early phase clinical trials with drug repurposing strategies demonstrated promising outcomes in patients with both rare and common treatment refractory advanced cancers. Despite clinical efficacy advancing drug repurposing strategies in the clinical trial trajectory beyond early phase studies has been challenging mainly due to lack of funding and interest from the pharmaceutical industry. In this review, we delineate our experience and challenges with drug repurposing strategies.

## INTRODUCTION

Despite the tremendous resources invested in anticancer therapy, cancer remains among leading causes of mortality worldwide [1]. Only 5% of oncology drugs entering Phase I clinical trials are ultimately approved, and drug development takes an average of 13 years at a cost as high as $1.8 billion [2]. The prolonged duration and enormous costs of the clinical trials required for regulatory approval by the U.S. Food and Drug Administration (FDA) emphasize the need for alternative strategies.

Relatively little attention is paid to utilizing existing drugs in novel combinations and regimens for enduring cancer indications. The major advantages of this “drug repurposing” approach are that the preclinical, pharmacokinetic, pharmacodynamic, and toxicity profiles of the drugs are already known and thus the repurposed regimens may rapidly translate into Phase II/III clinical studies. Drug repurposing also could reduce the costs of developing new drug therapies, especially when the patent protection expires, allowing generic manufacturing. Although drug repurposing and development of new combinations using existing agents are getting more attention [2], and multiple studies have shown their potential benefit in cancer care [3-8], the design, conduct, and most importantly funding of such studies remain major challenges. Here we discuss our experience with this approach in the Department of Investigational Cancer Therapeutics (Phase I Clinical Trials Program) at The University of Texas MD Anderson Cancer Center and the challenges facing the broader application of this approach.

### Drug repurposing strategies for cancer therapy in early phase clinical trials

Our department has been conducting broad early phase studies across disease types and molecular targets for the past 10 years. We have developed multiple clinical trials using a variety of repurposing strategies, and some of the approaches have resulted in very encouraging data. These trials were supported almost exclusively by institutional funds, and despite very promising results that often surpassed outcomes for emerging experimental anticancer agents, the lack of funding and other support have precluded further development. Some of these combinations are described here.

A Phase I clinical trial combining liposomal doxorubicin, bevacizumab, and temsirolimus (DAT) for patients with advanced cancers was designed to test the preclinical rationale that resistance to anthracyclines is driven through upregulation of hypoxia-inducible factor alpha (HIF-1α), which promotes angiogenesis and tumor survival. Thus inhibiting angiogenesis, such as with the VEGF inhibitor bevacizumab, may overcome anthracycline resistance. However, resistance to bevacizumab is also driven by upregulation of HIF-1α. Addition of temsirolimus, a potent inhibitor of mTOR and consequently HIF-1α, can overcome this resistance [9]. During the dose-escalation phase we noticed remarkable activity in several distinct tumor types, including metaplastic breast cancer (MpBC) [8] and gynecologic malignancies [6].

MpBC is a rare subtype of breast cancer that typically does not express estrogen/progesterone receptors or human epidermal growth factor receptor 2 (HER2) and thus usually is treated as a triple-negative breast cancer. However, MpBC patients experience more disease recurrence, and associated poorer overall survival, than other patients with triple-negative breast cancer and generally have a poor response to systemic therapy [10]. Therefore there is no standard therapy for MpBC. Despite the aggressive nature of this cancer, we observed 2 responses (1 complete [CR] and 1 partial response [PR]) in the 5 patients with treatment-refractory MpBC who received the DAT regimen on this trial. The CR was seen after 6 cycles of DAT, and the patient has been cancer free for more than 5 years and continues on maintenance therapy with the mTOR inhibitor as a single agent [8]. Encouraged by the promising beginning, we extended this cohort and observed objective responses in 5 of the 12 patients with MpBC (42%, 2 CR and 3 PR) and stable disease (SD) > 6 months in another 6 [50%] [6].

The DAT regimen was effective in diverse cancer types, including endometrial endometrioid carcinoma (6/15 [40%] PR), epithelial ovarian carcinoma (4/23 [17%] PR), and parotid gland adenocarcinoma (4/6 [67%] PR) [6]. On the basis of these promising outcomes, we have approached a cooperative group about conducting further clinical trials of the DAT regimen for MpBC. However, as of this writing, the study has not been opened. This contrasts starkly with the rapid approval of crizotinib for anaplastic lymphoma kinase gene–rearranged non-small cell lung cancer (NSCLC); crizotinib was approved approximately 2 years after its anticancer activity was initially observed [11].

Another example of an early phase clinical trial using a repurposing approach is our Phase I experience with the combination of mTOR inhibitor sirolimus and vorinostat in heavily pretreated patients with advanced cancer. Vorinostat is a histone deacetylase (HDAC) inhibitor that is approved by the FDA for treatment of cutaneous T-cell lymphoma and has a single-agent response rate of 4% in Hodgkin lymphoma refractory to standard therapies [12]. Novel HDAC inhibitors panobinostat or mocetinostat demonstrated objective response rates of 27% in the same patient population [13, 14]. Preclinical studies suggested that HDAC inhibitors, besides increasing histone acetylation, reduce the activity of AKT; however, that is circumvented by increasing mTOR activation through inhibition of LKB1 and adenine monophosphate–activated protein kinase. In preclinical studies, HDAC inhibitors were able to overcome mTOR-induced resistance [15], and this synergistic activity became the rationale for our Phase I clinical trial of sirolimus and vorinostat.

During the dose-escalation phase, we observed anticancer activity in hepatocellular carcinoma and perivascular epithelioid cell tumor (data not shown) and most notably in treatment-refractory Hodgkin lymphoma. The 28 patients with treatment-refractory Hodgkin lymphoma enrolled in the study had received a median of 6 prior therapies; 23 (82%) had undergone autologous stem cell transplantation and 7 (25%) allogeneic stem cell transplantation. Despite the poor responses to prior therapies, the overall response rate in the vorinostat/sirolimus trial was 57% (9/28 [32%] CR and 7/28 [25%] PR)[5] with tolerable toxic effects (dose interruptions only: 4/28 [25%], dose interruptions and modifications: 15/28 [54%]). Considering that patients with refractory Hodgkin lymphoma generally have a poor clinical outcome, the combination of sirolimus and vorinostat appears to be a promising treatment strategy.

Interestingly, similar activity was shown by Oki *et al* in a trial of the combination of everolimus and panobinostat in patients with relapsed or refractory lymphoma. In 14 patients with Hodgkin lymphoma, the overall response rate was 43% (6/14) and the CR rate 14% (2/14) [16]. Notably, the discontinuation rate due to intolerance was higher (43% [13/30]) than in our trial of sirolimus and vorinostat (4% [1/28]). The lower rate in our study may have been related to, among other factors, more flexible dosing and dose adjustments for sirolimus and vorinostat, which are both FDA approved (although not for Hodgkin lymphoma). Finally, the sirolimus and vorinostat regimen has a relatively favorable cost compared to some other novel FDA-approved therapies such as brentuximab; the estimated cost for 1 cycle of brentuximab is $13,683, while that for vorinostat/sirolimus is $7,696. The cost of the vorinostat/sirolimus regimen is likely to drop even further when the vorinostat patent protection expires in the near future, making this combination attractive in settings of more limited resources. Nevertheless, so far advancing this combination further in the clinical trials trajectory has been difficult.

A final example is a phase I clinical trial of bevacizumab in combination with an EGFR- or HER2-targeted agent. Preclinical data indicate that anti-VEGF treatment can augment response to HER2 or EGFR inhibition and increase apoptosis [17] and that dual blockade of tyrosine-kinase receptors with a monoclonal antibody at the extracellular domain and a tyrosine kinase inhibitor at the intracellular domain is effective [18]. These preclinical observations provided a rationale for combining anti-HER2 agents trastuzumab and lapatinib with bevacizumab in patients with metastatic HER2-positive breast cancer pretreated with a median of 7 regimens, including HER2-targeted therapies. Patients were able to tolerate the recommended FDA-approved doses of all 3 agents. Of the 26 patients enrolled, 1 [4%] had CR, 6 [23%] had PR, and 6 [23%] had SD ≥ 6 months [3]. Similarly, among 34 patients with NSCLC refractory to a median of 4 prior therapies who were treated with bevacizumab, erlotinib, and cetuximab, 4 [12%] had PR and 7 [21%] SD ≥ 6 months [4]. These combinations appear to have less favorable costs than other repurposing strategies mentioned; however, most of these compounds are nearing the end of their patent protection, and the costs are expected to drop significantly in the years to come. Unfortunately, we have been unable to secure adequate support to move these treatments along in the drug development pipeline.

### Clinical development challenges in rug repurposing strategies

**Figure 1:**
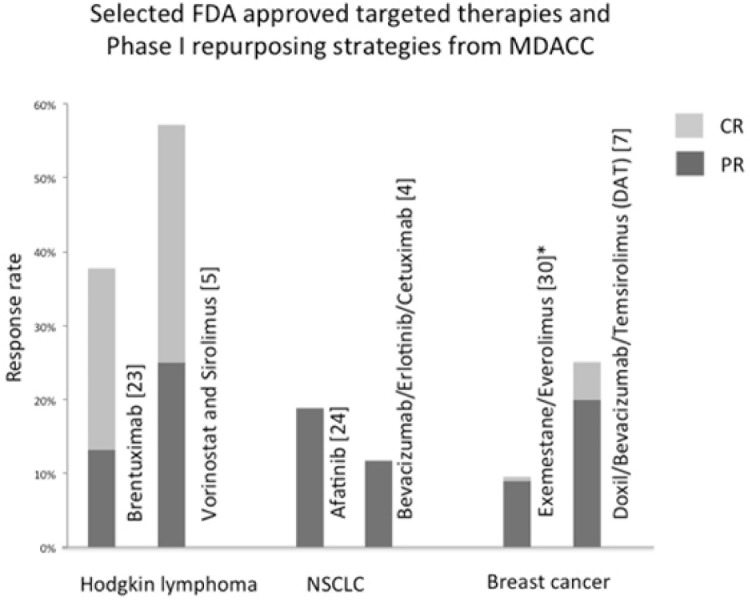
Overall response rate of selected FDA-approved novel targeted therapies (left) and repurposing-based combinations (right) in early phase clinical trials Numbers in parentheses are the citation of the clinical trial. All response rates shown are from Phase I trials, except *from Phase III trial data [30].

Historically, the expected response rate in Phase I trials with unselected populations of patients has ranged from 4% to 11% [19–22]. However, with recent advances in targeted therapies and molecular matching, the response rates in Phase I trials have ranged from 19% to 77%, and some agents that demonstrated high response rates are now FDA approved [23–28]. Even in heavily pretreated patients, drug repurposing combination regimens have yielded response rates ranging from 12% to 57% [3–8] (Figure 1).

Despite these promising results and the lower costs of existing agents, financial support for drug repurposing approaches has been lacking. In fact, the lower drug prices, short patent duration, and low return on investment are likely reasons that the pharmaceutical industry is not interested in investing in such clinical trials. Therefore, we propose that such efforts be more easily funded through federal sources such as National Cancer Institute and other agencies [29].

## CONCLUSIONS

Our experience has shown favorable outcomes with certain drug-repurposing strategies in cancer treatment, (Figure 1) which may reduce the cost and timeline for approval. However, this approach has several limitations. First, our studies were single institutional experience reviewing several investigator-initiated trials, non-randomized, dose-finding trials in which some of the enrolled patients may have received less then optimal dose. Second, our studies enrolled relatively small numbers of patients, often with diverse treatment-refractory advanced malignancies, which may have complicated data interpretation. Third, a substantial proportion of the patients had already been treated with drugs included in the novel combinations.

Lack of funding and interest from the pharmaceutical industry limits the clinical development trajectory. Potential solutions include support from peer-reviewed federal funding sources, which are centered more on patient and public health outcomes than commercial interests.
